# Observability of Complex Systems: Finding the Gap

**DOI:** 10.1038/s41598-017-16682-x

**Published:** 2017-11-29

**Authors:** J. D. Stigter, D. Joubert, J. Molenaar

**Affiliations:** Biometris, Department of Mathematical and Statistical Methods, Wageningen University and Research, Wageningen, 6708 PD The Netherlands

## Abstract

For a reconstruction of state and parameter values in a dynamic system model, first the question whether these values can be *uniquely* determined from the data must be answered. This structural model property is known as observability or, in case of parameter calibration only, *identifiability*. Testing a given model for observability is a well studied problem in the systems and control sciences. However, it is increasingly difficult, if not impossible, to address this property for large size models that, nowadays, are frequently used. We demonstrate the application of a recently developed algorithm that overcomes this problem and is remarkably efficient. As an illustration we show how an observability analysis for a Chinese Hamster Ovary Cell model (34 states, 117 parameters), a JAKSTAT signalling model (31 states, 51 parameters), and a MAP Kinase model (100 states, 88 parameters) can be established in a very short time.

## Introduction

Reconstructing the values of certain time varying variables *x*(*t*) or time-independent parameters *θ* for a given dynamic system model from measurement signals touches upon a fundamental question, namely ‘Is it actually possible to find the values of these unknowns, given a data-record of measurement signals?’ In his celebrated paper on mathematical systems theory, Kalman introduced the so-called observability property for models in state-space format. His definition of this structural model property (together with its dual form, i.e. structural controllability) is restricted to so-called linear-time-invariant (LTI) system models^1^. An LTI dynamic system model may represent, for example, a linear approximation of a large network of chemical reactions at a certain operation point ($\bar{x}$) that may be graphically summarized in a directed graph^2^. Given an input-output signal pair {*u*(*t*), *y*(*t*)} (where the input variables *u*(*t*) can be thought of as known signals, manipulated by the user, and the output variables *y*(*t*) are the dynamic measured response signals), both the unknown parameters *θ* and the internal states *x*(*t*) need to be reconstructed from this signal pair. Think, for example, of concentrations of chemicals that are involved in a reaction scheme: While only a subset of their concentrations can be measured, other concentrations of chemical species have to be deduced from the given data on the basis of a model and a so-called ‘state-observer’^3^.

Kalman showed that for LTI system models an observability rank test suffices to theoretically address the question whether the state *x*(*t*) can be reconstructed from the available data^1^. For the observability question for *both* unknown states and parameters, the Kalman observability test immediately poses a difficult problem: Once we have augmented the state vector *x*(*t*) with the model parameters *θ* to form a new state vector $$(\begin{array}{c}x(t)\\ \theta \end{array})$$, the system model becomes *non-linear*. This non-linearity introduces substantial difficulties in the observability analysis and the Kalman rank test can not be used straightforwardly. It took until the late 1970’s before this problem was actually understood in a satisfactory way using more advanced tools from the then emerging field of non-linear control theory^4–6^. The problem with these advanced tools, however, is that the actual computation involves so-called Lie-derivatives and Lie-brackets^6^. Computation of higher order brackets and/or Lie-derivatives is, in general, cumbersome for large system models leading to unacceptable computation times and memory requirements.

**Figure 1:**
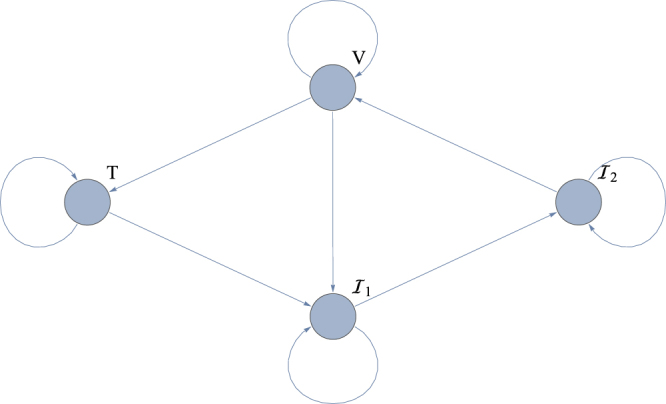
Directed graph summarizing the information flow in the Influenza model.

To provide context, we start with a simple non-linear example and then extend the approach we take to large non-linear system models. In a review paper^7^ several influenza models are analysed for identifiability of their parameters. The following 4-state model is an example of a target cell-limited influenza model with delayed virus production and reads

1 $$\frac{dT(t)}{dt}=-\beta \,T(t)\,V(t)$$

2 $$\frac{d{I}_{1}(t)}{dt}=\beta \,T(t)\,V(t)-k\,{I}_{1}(t)$$

3 $$\frac{d{I}_{2}(t)}{dt}=k\,{I}_{1}(t)-\delta \,{I}_{2}(t)$$

4 $$\frac{dV(t)}{dt}=p\,{I}_{2}(t)-c\,V(t)$$

where *T*(*t*) is the number of uninfected target (epithelial) cells, *I*_1_(*t*) the number of latent infected epithelial cells, not producing virus, *I*_2_(*t*) the number productively infected epithelial cells, and *V*(*t*) is the infectious viral titer which is the *only* variable measured. The parameters to be determined from the *V*(*t*) readings are *β*, *δ*, *c*, *k*, *p*. In addition to these model parameters, we also include the initial conditions *T*(0), *I*_1_(0), *I*_2_(0), *V*(0) as unknowns and analyse whether their values can be reconstructed from the *V*(*t*) readings. A directed graph of the influenza model (Fig. 1) shows that the information flow between the states *T*(*t*), *I*_1_(*t*), *I*_2_(*t*), and *V*(*t*) is good, meaning that the measured state *V*(*t*) receives inflow from all other states, either directly or indirectly, so that one would expect that all parameters should be identifiable. Using the implicit function method Miao *et al*. found that the parameter *p* in equation (4), however, cannot be identified from the sensor readings^7^. In the Supplemental Material 1.1 we show that this parameter is indeed totally correlated with the initial conditions *T*(0), *I*_1_(0), and *I*_2_(0), meaning that four (out of nine) parameters are completely undetermined in this specific experimental set-up. Of course, this negative result leads to quite a dramatic conclusion, namely: Whatever the number and quality of the viral titre data *V*(*t*) is, it is *impossible* to ascertain all unknowns from these data. Ignoring this insight one would, by applying one of the many available parameter estimation algorithms, arrive at results that are totally unreliable for the correlated unknowns in the model.

An enormous effort has been put into finding algorithms to assess the observability question for non-linear systems^8^. Most of these algorithms are *algebraic* observability tests that suffer from one important drawback: Due to their symbolic/algebraic nature, computer algebra systems must be used to calculate the derivatives of the output equation repeatedly and, while this is in many cases tractable for small scale system such as the influenza model, the symbolic computations become intractable for large scale systems in view of the computation time needed. Chappell *et al*.^9^ already noted this problem in the early nineties and, fairly recently, their conclusion was re-confirmed for the current state-of-the-art algorithms in this field^10^. Other, numerically based methods, such as the sensitivity based identifiability test have mainly been used to assess *practical* identifiability^11^, meaning that these methods are used to find which parameters can be estimated from *real* data that include noise-corrupted observations. If we assume perfect, noiseless data, *theoretical* observability addresses the question whether it is mathematically possible to find unique values for the unknowns in the model.

Liu *et al*.^12^ note in their recent and excellent review on the control principles of complex systems that control theory has unfortunately not been very successful in answering the theoretical observability question for complex systems. The currently best available *symbolic* algorithm is due to Sedoglavic^13^ and has been applied to large models by Anguelova^14^. In Liu’s observability paper it was used to verify a novel graph-theoretical based observability test^2^. This novel test comprises a matching algorithm and it is capable of quickly finding the root-compartments in a directed graph of the dynamic model. From these root compartments sensors must then be selected for the system to become observable. The graph-theoretical approach uses as its only input the adjacency graph of a given network and there is no direct reference to the algebraic relations that connect the state *x*(*t*) and the parameters *θ*. In addition, Liu’s question only includes observability of initial *states* in his analysis, while the parameters that are involved in the interactions are assumed known or, better, only deemed relevant for the analysis in the sense that their values are either zero (there is no connection between two nodes *i* and *j*) or non-zero (there is a connection between two nodes *i* and *j*).

## Results

Yet, we claim there is a way out of the cul-de-sac of finding an *algebraic* answer to the structural observability question. In this escape the so-called parametric output sensitivity matrix plays a crucial role. To explain this role, consider the dynamic behaviour of the general non-linear state space model

5 $$\frac{dx(t)}{dt}=f(x(t),u(t),\theta )$$

6 $$x\mathrm{(0)}={x}_{0}$$

7 $$y(t)=h(x(t),u(t),\theta )$$

with (5) and (7) the dynamical model and measurement equations, respectively, *dim*(*x*) = *n*, *dim*(*u*) = *r*, and *dim*(*θ*) = *p*. If we include the initial conditions *x*_0_ as *additional parameters* in the parameter vector *θ*, we get a *q* = (*n* + *p*)-dimensional parameter vector *θ*. For this augmented parameter vector *θ* the sensitivity dynamics become:

8 $$\frac{d{x}_{\theta }(t)}{dt}=\frac{\partial f}{\partial x}\,{x}_{\theta }(t)+\frac{\partial f}{\partial \theta }$$

9 $${y}_{\theta }(t)=\frac{\partial h}{\partial x}\,{x}_{\theta }(t)+\frac{\partial h}{\partial \theta }$$

with *x*_*θ*_ = (∂*x*)/(∂*θ*) and *y*_*θ*_ = (∂*y*)/(∂*θ*). The matrix *x*_*θ*_ has dimensions (*n* × *q*). For the extra initial condition parameters *x*_0_ the sensitivities are initialized as *x*_*θ*_(0) = *I*_*n*_, i.e. the identity matrix, while *x*_*θ*_(0) = *O*_*n* × *p*_ (a zero-matrix), for the original system parameters. Note that the sensitivity equation (8) constitutes *q linear, time-varying, systems* that run in parallel and whose seed of dynamics originates from the original system model (5). Each of these *q* sensitivity systems generates its own dynamics for one particular parameter *θ*_*i*_, *i* = 1, …, *p*, *p* + 1, …, *q* = *n* + *p*. The parametric output sensitivities *y*_*θ*_(*t*) can now be calculated by integrating (5) and (8) and substituting the solution into (9). This allows construction of the well known *sensitivity matrix* ^15,16^

10 $$S({t}_{0},\ldots ,{t}_{N},\theta )=(\begin{array}{ccc}\frac{\partial {y}_{1}({t}_{0})}{\partial {\theta }_{1}} & \ldots  & \frac{\partial {y}_{1}({t}_{0})}{\partial {\theta }_{q}}\\ \vdots  &  & \vdots \\ \frac{\partial {y}_{m}({t}_{0})}{\partial {\theta }_{1}} & \ldots  & \frac{\partial {y}_{m}({t}_{0})}{\partial {\theta }_{q}}\\ \vdots  &  & \vdots \\ \frac{\partial {y}_{1}({t}_{N})}{\partial {\theta }_{1}} & \ldots  & \frac{\partial {y}_{1}({t}_{N})}{\partial {\theta }_{q}}\\ \vdots  &  & \vdots \\ \frac{\partial {y}_{m}({t}_{N})}{\partial {\theta }_{1}} & \ldots  & \frac{\partial {y}_{m}({t}_{N})}{\partial {\theta }_{q}}\end{array})$$

This matrix can be thought of as a series of “snapshots” of the sensitivity dynamics *y*_*θ*_(*t*) that have been stacked vertically, one for each time instant *t*_*i*_. When moving downwards from top to bottom in the above matrix, we are watching a stroboscopic film, each frame consisting of a snapshot of the sensitivity dynamics at time instant *t*_*i*_. A *local* (or at-a-point) structural observability test may now be formulated that is based on a rank-test of the sensitivity matrix^17–19^. A full rank sensitivity matrix is a *sufficient* condition for observability^7^. This fact can be re-phrased into its contrapositive as: If a system is *not* observable, then the matrix *S*(*t*_0_, …, *t*_*N*_, *θ*) *must* be rank deficient.

A well-known tool to assess the rank of a given matrix is the singular value decomposition^20^ (SVD) that allows the matrix to be written as a sum of equally sized matrices that decrease in dominance (or singular value *σ*_*i*_) as

11 $$S({t}_{0},\ldots ,{t}_{N},\theta )={u}_{1}\,{\sigma }_{1}\,{v}_{1}^{T}+\ldots +{u}_{q}\,{\sigma }_{q}\,{v}_{q}^{T}$$

Having this decomposition of a given sensitivity matrix *S*(*t*_0_, …, *t*_*N*_, *θ*) available for a value of *θ*, the observability question can be rephrased to one that asks for the nullity of the *last* singular value *σ*_*q*_. Indeed, if for some *k* ≤ *q*, *σ*_*k*_ = … = *σ*_*q*_ = 0, we know that *S*(*t*_0_, …, *t*_*N*_, *θ*) is rank deficient and finding unique values of all parameters in the model is very likely to fail. Hunting down one or several zero singular values is therefore an important mission in answering the question of a lack of observability. In fact, detecting zero singular values can be considered as part 1 of our strategy. In the supplemental material we demonstrate that there is, in addition, a part 2, namely the symbolic verification of a lack of identifiability, once we have detected this on the basis of a SVD of the sensitivity matrix (10).

Recent work^19,21^ shows that observability is decided upon by setting a *threshold* on the eigenvalues of the Fisher Information Matrix (FIM) below which a model is considered not identifiable. A proxy of the FIM that has exactly the same rank as (10) is simply calculated by multiplying the transposed of the sensitivity matrix with itself. A known source of inaccuracy is that a rank test on the FIM is not as precise as a rank test on the original sensitivity matrix (10), see also Moore’s paper where this is already noted^22^. Another source of inaccuracy is that only one trajectory (or simulation) of the model is considered, corresponding to one value for the parameters in the vector *θ*. Here we *combine* several trajectories for different values of the parameter vector *θ* and we will demonstrate (see Supplementary Information 1.2) that this leads to a dramatic improvement in the accuracy of a zero-singular-value-detection. In addition, a wealth of information can be deduced by first looking at the singular vectors {*v*_*i*_, *i* = *k*, …, *q*} for which *σ*_*i*_ = 0, i.e. the basis of the nullspace of *S*(*t*_0_, …, *t*_*N*_, *θ*)^18,23^. We demonstrate that using this information leads to a tremendous simplification and allows a tractable symbolic computation to be performed that further tests the SVD-detected lack of observability and validates these results.

### The nullspace basis vector that shows the correlations

**Figure 2:**
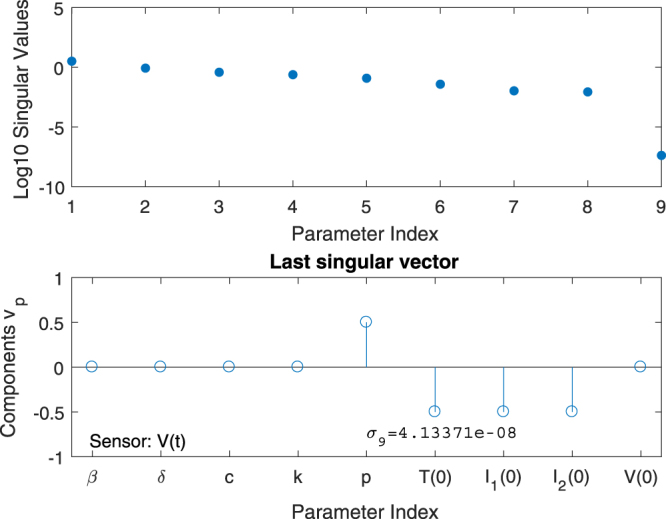
Observability signature for the Influenza model: Since there are nine parameters to be determined, there are nine columns in the matrix *S*(*t*_0_, …, *t*_*N*_, *θ*). An SVD of this matrix then yields nine singular values *σ*_1_, …, *σ*_9_. These singular values are presented in the top graph on a logarithmic scale. Note the sudden *gap* between *σ*_8_ and *σ*_9_ of approximately 3.5 decades on the logarithmic scale, indicating a true zero for the last singular value and, hence, a possible lack of observability. The bottom graph clearly shows that the parameters *q*, *T*(0), *I*_1_(0), and *I*_2_(0) are involved in this total correlation, suggesting that there is an algebraic relationship between these parameters that deems the model un-identifiable.

Consider the case that one singular value is zero, i.e. *σ*_*q*_ = 0. The corresponding right singular vector *v*_*q*_ in (11) provides detailed information on the parameters that are correlated and cause the model to be unidentifiable. Indeed, the entries in *v*_*q*_ clearly show which of the columns in the sensitivity matrix are linearly dependent and we therefore only need to inspect the non-zero entries in this vector to find out *which* parameters are correlated. Note that this is not necessarily a pairwise correlation between two parameters but, rather, a complete list of those parameters that are involved in a total correlation. As an example consider, again, the Influenza case study. Calculating a SVD of the sensitivity matrix for random values of *θ* ^*i*^ yields what we term as the *observability signature* of the given model and experimental configuration^23^. In general, the observability signature is summarized in two graphs, namely (i) a graph of the *q* singular values {*σ*_*i*_, *i* = 1, …, *q*} of the sensitivity matrix (10), in decreasing order, as a function of their index *i*, and (ii) a graph of the last right-singular vectors {*v*_*i*_, *i* = *k*, …, *q*} in (11) that correspond to zero singular values with, on the horizontal axis, the vector entry index *l* = 1, …, *q* and on the vertical axis the corresponding values in *v*_*i*_. In Fig. 2 a graph of the nine entries of the right singular vector *v*_9_ for the influenza model is presented. Its corresponding singular value *σ*_9_ was found in the order ${\mathscr{O}}{\mathrm{(10}}^{-8})$ and, when comparing this to the other 8 singular values, one can observe a clear *gap* between *σ*_8_ and *σ*_9_ of approximately 3.5 decades on the logarithmic scale (Fig. 2). This is typical for a rank deficient sensitivity matrix. From Fig. 2, bottom panel, we further conclude that *four* out of nine parameters are involved in a total correlation. These are the parameters *p*, *T*(0), *I*_1_(0), and *I*_2_(0). Knowing this correlation between these specific parameters, we may re-parametrise the model on the basis of a simplified symbolic computation (see Supplementary Information 1.1). Since the sensitivity of the output with respect to the remaining 5 parameters is *not* present in the SVD-detected nullspace, these parameters can, in principle, be identified from the sensor readings.

### Pushing the Limits: Complex Systems

An immediate question is whether the idea of a rank test of *S*(*t*_0_, …, *t*_*N*_, *θ*) can be scaled up to large simulation models with many states and parameters in their structural equations. The results we present in this paper demonstrate that the answer is definitely positive. This success stems for a substantial part from the SVD algorithm in combination with an accurate integration of equations (5–8). To guarantee a precise solution of the sensitivity equations we employed the use of complex derivatives^24^ that allow a very accurate computation of the Jacobi matrices $\frac{\partial f}{\partial x}$ and $\frac{\partial f}{\partial \theta }$ in (8), thereby decreasing the absolute tolerance on the sensitivities *y*_*θ*_(*t*) to 10^−20^. In addition, combining several sensitivity matrices for different parameter values also leads to a substantial improvement of the accuracy on a zero-singular-value detection as demonstrated in Supplementary Information 1.2. We have, until now, not seen a case yet in which the algorithm failed to demonstrate a lack of observability for models that, in the literature, are known to be unobservable. Our findings are substantiated with the following three large case studies, namely (i) The Chinese Hamster ovary cell model, (ii) the JAKSTAT model, and (iii) the MAP-Kinase model. These examples were chosen since the first two are the largest ones available in the current literature on local structural identifiability. Since algebraic/symbolic computations have been performed for these two large case studies, our results can therefore directly be compared. Furthermore, the third case study is a good demonstration of Liu’s conjecture on the observability of complex systems^12^.

#### Chinese Hamster Ovary cell model

**Figure 3:**
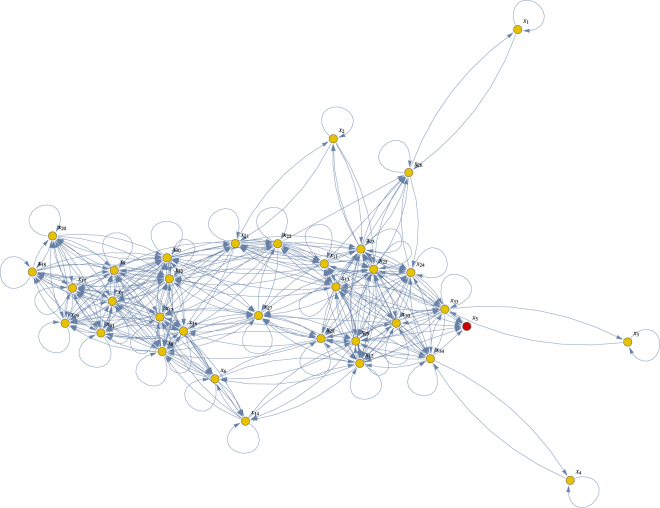
Directed graph Chinese Hamster case: The 34 nodes in this graph correspond to the 34 states *x*_*i*_(*t*), *i* = 1, …, 34. An arrow from node *x*_*i*_ to node *x*_*j*_ means that the *j* ^*th*^ differential equation in the model contains a term with the state variable *x*_*i*_(*t*) in it. Self-loops show that most nodes also influence their own dynamics. Finally, the connected nodes are coloured in yellow and red so that one can easily see that node *x*_5_ is a sink with no outgoing arrows.

The first large-scale example model describes the dynamic behaviour of 34 metabolites in three compartments (fermenter, cytosol, and mitochondria) of a Chinese Hamster Ovary cell. Thirteen of these metabolites can be measured directly, corresponding to 13 measurable states in the output equation (7), namely *x*_1_, *x*_2_, *x*_3_, *x*_4_, *x*_5_, *x*_11_, *x*_13_, *x*_15_, *x*_21_, *x*_27_, *x*_29_, *x*_30_, and *x*_32_. For exact details we refer to the paper^25^ that includes this example as one of a series of benchmark problems for structural identifiability analysis. This non-linear model has a total of 117 system parameters and 34 states in its structural equations. In Fig. 3 a directed graph is presented that summarizes the exact wiring diagram of these 34 states. Assuming the initial conditions are also unknowns, there are 117 + 34 = 151 parameters to be identified from the output signals. This identifiability case study is considered “very challenging”, since the symbolic computations to address identifiability for this model are so enormous that it takes many hours (if not days) to complete this task. These symbolic computer algebra results clearly show this model is *non-identifiable* ^25^.

**Figure 4:**
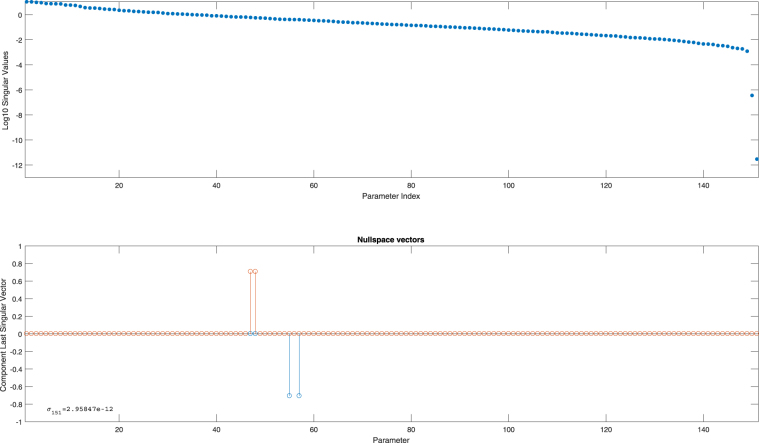
Observability signature Chinese Hamster model. The top graph shows all singular values for the 151 parameters in the model (including the parametrized initial conditions). A gap is clearly visible, indicating a lack of identifiability. The bottom graph shows the nullspace vectors for the sensitivity matrix corresponding to the two smallest singular values (order 10^−12^).

In Fig. 4 the observability signature for this large model is presented in two sub-plots. Generating the observability signature is *a matter of seconds* and only comprises a number of model simulations. Figure 4 immediately shows the typical gap in the singular values, indicating a possible lack of identifiability. There are two singular values detected as zero in this particular case and these correspond to two groups of correlated parameters. The “zeroness” of these last two singular values is even more apparent when looking at the nullspace graph of the last singular vectors ${v}_{150}$ and ${v}_{151}$. Clearly there are four parameters involved in the correlations and, since two zero singular values are detected, these four parameters can be classified into two groups of two correlated parameters each (see Supplementary Information 1.2).

#### JAKSTAT model

**Figure 5:**
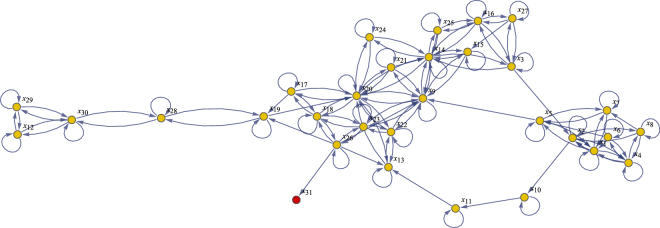
Directed graph JAKSTAT model: The 31 nodes in this graph correspond to the 31 states *x*_*i*_(*t*), *i* = 1, …, 31. The connected nodes are coloured in yellow and red so that one can easily see that node ${x}_{31}$ is the only one-node-sink with no outgoing arrows.

**Figure 6:**
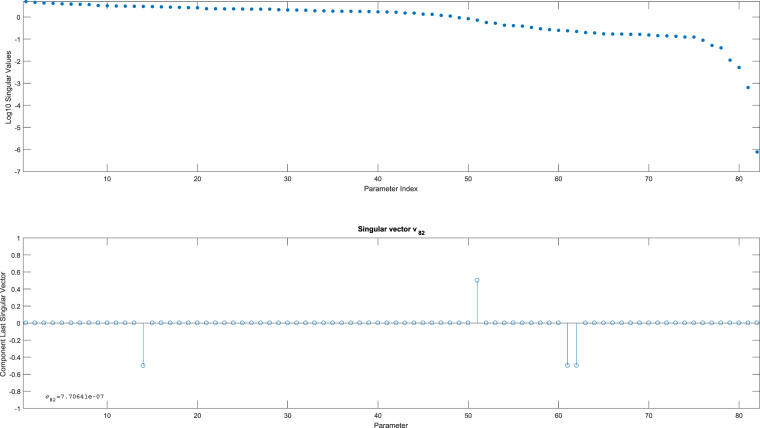
Observability signature JAK-STAT model for a concatenation of five trials for the sensitivity matrix (10) when *x*_10_ and *x*_11_ are *not* in the sensor set. The top graph shows all singular values for the 82 parameters in the model (including the parametrized initial conditions). The gap indicates a lack of observability and the bottom graph summarizes the parameters involved, namely ${\theta }_{14},{\theta }_{51},{x}_{10}\mathrm{(0),}\,{x}_{11}\mathrm{(0)}$. A symbolic verification confirmed a lack of observability for this particular sensor set.

In her paper on minimal output sets that allow identification of all parameters in a model, Anguelova checks observability of the JAKSTAT model (summarized in a directed graph in Fig. 5) after first sifting out so-called translation and scaling (Lie-)symmetries. The final observability test is performed with Sedoglavic’ algorithm and takes most of the computation time^14^. This well-known signalling model^26^ comprises 31 states and 51 parameters and was shown to be un-identifiable. We computed in roughly 2 seconds the observability signature (using five trial values *θ* ^*i*^) for the JAK-STAT model for a sensor set that does *not* include the states *x*_10_ and *x*_11_ (these two states are responsible for a lack of identifiability). The result is summarized in Fig. 6. Again, we observe a clear gap in the singular values, and the bottom panel in this figure immediately shows that parameters ${\theta }_{14},{\theta }_{51},{x}_{10}\mathrm{(0),}\,{x}_{11}\mathrm{(0)}$ are responsible for this. This result verifies Anguelova’s findings^14^.

#### MAP Kinase model

**Figure 7:**
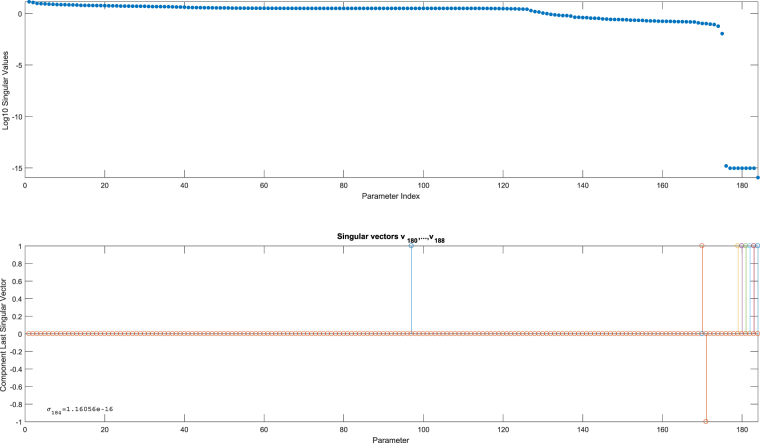
Observability signature Map Kinase model. The top graph shows all singular values for the 184 parameters in the model (including the parametrized initial conditions). Again, a gap is clearly visible, indicating a lack of identifiability if the one-node-sinks *x*_13_, *x*_86_, *x*_87_, *x*_95_, *x*_96_, *x*_97_, *x*_98_, *x*_99_, and *x*_100_ are not included in the sensor set. The bottom graph shows the nullspace vector that indicates precisely these nodes.

**Figure 8:**
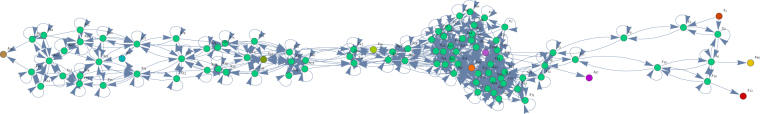
Directed graph MAP Kinase model. Connected components are labelled with colors. There are nine one-node-sinks observable.

In our third example we analyse the well-known MAP Kinase model^27^ by Schoeberl *et al*.. Details of this model have been widely discussed in the literature. The number of states is 100, while there are 84 system parameters to be identified. Including the initial conditions as unknowns we have 184 parameters to be identified from the sensor readings. For this model we first applied our method using various sensor combinations, assuming that the states could be measured directly (which is not realistic in practice). The sensors were assigned as a random permutation of 50 out of 100 possible direct state measurements and 100 randomly chosen sensor sets were then analysed. In all cases gaps were observable, see Fig. 7. These gaps correspond to state-initial-condition-parameters *x*_0_ only. After further inspection, these states were all found to be *sinks* in the network structure that have no outgoing arrows. For the initial condition of such a sink-node to be estimated, one must include it as a sensor because otherwise, of course, no information on its corresponding parameter in the parameter vector *x*_0_ can be inferred from the other sensors. Put differently, our approach results in an alternative method of finding the sinks of a network from which no information of their initial condition can be received on the sensor readings if these nodes are not part of the sensor set. In fact, these results confirm Liu’s conjecture^2^ that, in order to have an observable model, one has to include one of the sensors in each root compartment. For the MAP Kinase model this means that at least sensors *x*_13_, *x*_86_, *x*_87_, *x*_95_, *x*_96_, *x*_97_, *x*_98_, *x*_99_, and *x*_100_ have to be in the sensor set in order to identify (theoretically) all unknowns in the model. This result is completely in tune with the directed graph presented in Fig. 8 where exactly these nodes are highlighted in colour as one-node-sinks of the directed graph associated with the MAP Kinase model.

## Discussion

The results in this paper show that observability of non-linear large scale models can be achieved in a short computation time that is orders of magnitudes smaller than what is currently common-practice using algebraic observability tests. The avenue we followed opens up interesting perspectives for future research. For example, it is well known that structural controllability is the dual version of the structural observability problem discussed here. Extending the analysis to local controllability is therefore within easy reach, and will be the subject of further investigation. We note that preliminary results on this front are promising.

Finally, we have restricted our examples to complex biological systems but, of course, the methodology can be easily used in other applications as well. For example, observability is an important topic in aeronautics^28^ and helps the engineer to decide on optimal design and sensor placement for position and velocity estimation of space vehicles. Another application is fluid dynamics in which exactly the same type of questions need to be addressed^29^. These are just two of the many examples that can be analysed in detail with the algorithm we have explained and demonstrated in the above.

## Electronic supplementary material

Supplementary Information
