# Supplementary material for: Observability of Complex Systems: Finding the Gap
Source: Sci Rep. 2017 Nov 29;7:16566. doi: 10.1038/s41598-017-16682-x (PMC5707395; doi:10.1038/s41598-017-16682-x)
Supplement: Supplementary file 1 — Supplementary Information [file 41598_2017_16682_MOESM1_ESM.pdf]

Observability of Complex Systems: Finding the Gap

J.D. Stigter, D. Joubert, and J. Molenaar

1 Supplemental Material

1.1 Symbolic computation nullspace Influenza model

The observability signature in figure 2 (main text) gives an important lead in finding a possible re-parametrisation of the model that leaves the output invariant and, herewith, shows explicitly that there is a lack of observability. First, we look at the Lie-derivatives of the output signal $V(t)$ to find a generating series:

$$\begin{aligned}
\mathscr{L}_f^0 h &= V(0) && (1)\\
\mathscr{L}_f^1 h &= I_2(0)\,p - V(0) && (2)\\
\mathscr{L}_f^2 h &= -c\,I_2(0)\,p - \delta\,I_2(0)\,p + I_1(0)\,k\,p + c^2\,V(0) && (3)\\
\mathscr{L}_f^3 h &= (c+\delta)\,(\delta\,I_2(0) - I_1(0)\,k)\,p + c^2\,(I_2(0)\,p - c\,V(0) + \ldots \\
&\quad k\,p\,(-I_1(0)\,k + \beta\,T(0)\,V(0)) && (4)
\end{aligned}$$

These coefficients are now used to find the Jacobi matrix with respect to the correlated parameters $p, T(0), I_1(0), I_2(0)$. This evaluates to a (4×4) matrix $G(\theta)$:

$$\begin{pmatrix} 0 & 0 & 0 & 0 \\ I_2(0) & 0 & 0 & p \\ -cI_2(0) - \delta I_2(0) + I_1(0)k & 0 & kp & -(c+\delta)p \\ I_2(0)c^2 + (c+\delta)(\delta I_2(0) - I_1(0)k) + k(\beta T(0)V(0) - I_1(0)k) & \beta kpV(0) & -k(c+\delta+k)p & \left(c^2+\delta(c+\delta)\right)p \end{pmatrix} \tag{5}$$

whose nullspace is

$$\mathscr{N}(G(\theta)) = \begin{pmatrix} -\frac{p}{I_2(0)} & \frac{T(0)}{I_2(0)} & \frac{I_1(0)}{I_2(0)} & 1 \end{pmatrix} \tag{6}$$

Adding more rows to $G(\theta)$ by calculating more Lie-derivatives does not destroy the nullspace. Revealing the structure of the nullspace $\mathscr{N}(G(\theta))$ symbolically yields more than just knowledge of the existence of a non-trivial nullspace. We can interpret equation (6) as: A linear combination of the sensitivities $y_p, y_{T(0)}, y_{I_1(0)}$, and $y_{I_2(0)}$ is apparently equal to zero and the coefficients of this linear combination can be found in the nullspace basis vector. In other words, we have

$$-\frac{p}{I_2(0)}\,y_p(t) + \frac{T(0)}{I_2(0)}\,y_{T(0)}(t) + \frac{I_1(0)}{I_2(0)}\,y_{I_1(0)}(t) + y_{I_2(0)}(t) = 0 \tag{7}$$

Assume now we are in a regular point $\bar{\theta}$, meaning that if we sample parameter vectors θ^i in an open neighbourhood of $\bar{\theta}$ and evaluate the rank of the sensitivity matrix $S(t_0,\ldots,t_N,\theta^i)$, then its rank does not change. Since observability is a *structural property*, the relation (7) holds for all different parameter values θ^i, and we are therefore allowed to *combine* the sensitivity matrices $S(t_0,\ldots,t_N,\theta^i)$ into one overall matrix by concatenation of the columns in $S(t_0,\ldots,t_N,\theta^i)$. This observation leads to quite a dramatic improvement of the accuracy in obtaining the zero singular values. The increase in accuracy is demonstrated in section 1.2.

Finally, knowing the nullspace structure symbolically allows a re-parametrisation of the model to be calculated so that a dynamic model with one parameter less, and having the same output behaviour, can be found[1]. For the influenza model, the new states that remove parameter p from the dynamics are:

$$\tilde{T}(t) = p\,T(t),\ \tilde{I}_1(t) = p\,I_1(t),\ \tilde{I}_2(t) = p\,I_2(t) \tag{8}$$

A second crucial advantage we demonstrate in this simple example is that knowledge of the correlated parameters via the SVD analysis leads to a *substantially* simplified symbolic computation since not all nine columns of the Jacobi matrix (5) need to be generated. Hence, to demonstrate rank deficiency of the Jacobi matrix for the Influenza model, one only needs to differentiate the output function 3 times in this particular example and this already completes a proof of a lack of identifiability for this particular model[2].

1.2 Symbolic computation nullspace Chinese Hamster Ovary model

For the Chinese Hamster Ovary (CHO) model the observability signature in figure 4 (main text) shows clearly that (i) there are two zero singular values detected as zero, and (ii) four parameters, namely p_{47}, p_{48}, p_{55}, and p_{57} are participating in two correlations. The differential equations that include these four parameters correspond to the states $x_{12}, x_{23}, x_{24}, x_{25}, x_{26}, x_{27}, x_{28}$, and x_{29}. Trying out different sensor combinations for the CHO model, it was found that the choice of which states were observed (from the 34 possibilities) did *not* matter. In all cases the same nullspace was detected, including two zero singular values.

To demonstrate the accuracy in finding the two zero-singular-values, we performed several model simulation runs and compared the SVD results between (i) a vertical concatenation of five matrices $\{S(t_0,\dots,t_N,\theta^i), i = 1,\dots,5\}$ and (ii) the SVD results for only one simulation that yields $S(t_0,\dots,t_N,\theta^1)$. From supplementary figure 1 it is immediately clear that using only one trial value gives false positives, i.e. zero singular values are detected that are not truly zero. This could have occurred because of a wrong choice of θ^1, meaning that our sensitivity matrix is evaluated at a non-regular point. Yet, the chances of choosing such a point are really very small and this lead us to conclude that more than one trial is needed. Once five trials are combined into one overall sensitivity matrix, the false positives vanish immediately and what is left are two true zero singular values. To validate these two zero singular values, we continued our analysis with a symbolic computation. Hereto, we calculated the Lie-derivatives for the sensor set that includes all the previously mentioned states and found the following Jacobi matrix, where columns 1–4 correspond to parameters p_{47}, p_{48}, p_{55}, and p_{57}, respectively:

$$\begin{pmatrix} 0 & 0 & 0 & 0 \\ 0 & 0 & 0 & 0 \\ 0 & 0 & 0 & 0 \\ 0 & 0 & 0 & 0 \\ 0 & 0 & 0 & 0 \\ 0 & 0 & 0 & 0 \\ 0 & 0 & 0 & 0 \\ 0 & 0 & 0 & 0 \\ 0 & 0 & -\log(x_{27}(0)) & \log(x_{27}(0)) \\ \log(x_{11}(0)) & -\log(x_{11}(0)) & \log(x_{27}(0)) & -\log(x_{27}(0)) \\ \log(x_{11}(0)) & -\log(x_{11}(0)) & 0 & 0 \\ -\log(x_{11}(0)) & \log(x_{11}(0)) & -\log(x_{27}(0)) & \log(x_{27}(0)) \\ -\frac{\log(x_{11}(0))}{2} & \frac{\log(x_{11}(0))}{2} & 0 & 0 \\ 0 & 0 & -\log(x_{27}(0)) & \log(x_{27}(0)) \\ 0 & 0 & \log(x_{27}(0)) & -\log(x_{27}(0)) \\ 0 & 0 & \log(x_{27}(0)) & -\log(x_{27}(0)) \end{pmatrix} \tag{9}$$

The nullspace for the above matrix can easily be calculated as

$$\mathcal{N}(G(\theta)) = \begin{pmatrix} 0 & 0 & 1 & 1 \\ 1 & 1 & 0 & 0 \end{pmatrix} \tag{10}$$

This result clearly shows that there are two separate groups of parameters in the observed correlations, namely p_{47} and p_{49} as the first group, and p_{55} and p_{57} as the second group.

1.3 Algebraic specification of the models

In the following the ordinary differential equations for each of the three large case study models are summarized as a reference.

1.3.1 Chinese Hamster Ovary model

```
d/dt(x1) = p108*log(x26) - p107*log(x1) - 1
d/dt(x2) = p65*log(x21) - p67*log(x2) + p66*log(x23) - p68*log(x25) + 1
d/dt(x3) = p110*log(x33) - p109*log(x3) - 1
d/dt(x4) = p112*log(x34) - p111*log(x4) - 1
d/dt(x5) = (x10*x12*x13*x24*x25*x29*x33*x34*(1/p1 + 1)*(1/p2 + 1)*(1/p3 + 1)*(1/p4
    + 1)*(1/p5 + 1)*(1/p6 + 1)*(1/p7 + 1)*(1/p8 + 1))/((x12/p3 + 1)*(x10/p7 + 1)*(
    x13/p8 + 1)*(x24/p1 + 1)*(x25/p2 + 1)*(x29/p6 + 1)*(x33/p4 + 1)*(x34/p5 + 1))
d/dt(x6) = p9*log(x8) - p11*log(x6) + p10*log(x9) - p12*log(x7) - p18*log(x6) + p19
    *log(x8) - p17*log(x15) + p20*log(x14) - p21*log(x16) - p96*log(x6) + p94*log(
    x12) + p93*log(x14) - p113*log(x6) - p95*log(x29) + p114*log(x12)
d/dt(x7) = p9*log(x8) - p11*log(x6) + p10*log(x9) - p12*log(x7) - p23*log(x7) + p25
    *log(x9) + p26*log(x9) - p29*log(x7) - p22*log(x16) + p24*log(x15) - 2*p37*log(
    x7) + p27*log(x19) - p30*log(x17) + 2*p39*log(x8) + p28*log(x20) - p31*log(x18)
    + 2*p40*log(x9) - 2*p36*log(x15) - 2*p38*log(x21) + 2*p42*log(x30) - 2*p41*log(
    x32) - p75*log(x8) - p76*log(x7) + p81*log(x9) - p77*log(x19) + p80*log(x16) +
    p82*log(x17) + p84*log(x15) - p78*log(x31) - p79*log(x32) + p83*log(x30) - 2
d/dt(x8) = p11*log(x6) - p9*log(x8) - p10*log(x9) + p12*log(x7) + p18*log(x6) - p19
    *log(x8) + p17*log(x15) - p20*log(x14) + p21*log(x16) + p37*log(x7) - p39*log(x8
    ) - p40*log(x9) + p36*log(x15) + p38*log(x21) - p42*log(x30) + p41*log(x32) -
    p75*log(x8) - p76*log(x7) + p81*log(x9) - p77*log(x19) + p80*log(x16) - p89*log(
    x8) + p82*log(x17) + p84*log(x15) + p92*log(x16) - p78*log(x31) - p79*log(x32) +
     p83*log(x30) - p90*log(x27) + p91*log(x28) - 1
d/dt(x9) = p11*log(x6) - p9*log(x8) - p10*log(x9) + p12*log(x7) + p23*log(x7) - p25
    *log(x9) - p26*log(x9) + p29*log(x7) + p22*log(x16) - p24*log(x15) + 2*p37*log(
    x7) - p27*log(x19) + p30*log(x17) - 2*p39*log(x8) - p28*log(x20) + p31*log(x18)
    - 2*p40*log(x9) + 2*p36*log(x15) + 2*p38*log(x21) - 2*p42*log(x30) + 2*p41*log(
    x32) + p75*log(x8) + p76*log(x7) - p81*log(x9) + p77*log(x19) - p80*log(x16) -
    p82*log(x17) - p84*log(x15) + p78*log(x31) + p79*log(x32) - p83*log(x30) + 2
d/dt(x10) = p13*log(x12) - p15*log(x10) + p14*log(x13) - p16*log(x11) - (120*x10*
    x12*x13*x24*x25*x29*x33*x34*(1/p1 + 1)*(1/p2 + 1)*(1/p3 + 1)*(1/p4 + 1)*(1/p5 +
    1)*(1/p6 + 1)*(1/p7 + 1)*(1/p8 + 1))/((x12/p3 + 1)*(x10/p7 + 1)*(x13/p8 + 1)*(
    x24/p1 + 1)*(x25/p2 + 1)*(x29/p6 + 1)*(x33/p4 + 1)*(x34/p5 + 1)) + 1
d/dt(x11) = p13*log(x12) - p15*log(x10) + p14*log(x13) - p16*log(x11) - p33*log(x11
    ) + p35*log(x13) - p32*log(x22) + p34*log(x21) - p85*log(x11) + p88*log(x13) -
    p98*log(x11) + p97*log(x13) - p86*log(x30) + p87*log(x32) + (1260*x10*x12*x13*
    x24*x25*x29*x33*x34*(1/p1 + 1)*(1/p2 + 1)*(1/p3 + 1)*(1/p4 + 1)*(1/p5 + 1)*(1/p6
     + 1)*(1/p7 + 1)*(1/p8 + 1))/((x12/p3 + 1)*(x10/p7 + 1)*(x13/p8 + 1)*(x24/p1 +
    1)*(x25/p2 + 1)*(x29/p6 + 1)*(x33/p4 + 1)*(x34/p5 + 1))
d/dt(x12) = p15*log(x10) - p13*log(x12) - p14*log(x13) + p16*log(x11) - p54*log(x12
    ) - p58*log(x10) + p53*log(x23) + p51*log(x28) + p52*log(x29) - p56*log(x25) -
    p55*log(x27) + p57*log(x27) + p96*log(x6) - p94*log(x12) - p93*log(x14) + p113*
    log(x6) + p95*log(x29) - p114*log(x12) - (240*x10*x12*x13*x24*x25*x29*x33*x34
    *(1/p1 + 1)*(1/p2 + 1)*(1/p3 + 1)*(1/p4 + 1)*(1/p5 + 1)*(1/p6 + 1)*(1/p7 + 1)
    *(1/p8 + 1))/((x12/p3 + 1)*(x10/p7 + 1)*(x13/p8 + 1)*(x24/p1 + 1)*(x25/p2 + 1)*(
    x29/p6 + 1)*(x33/p4 + 1)*(x34/p5 + 1))
d/dt(x13) = p15*log(x10) - p13*log(x12) - p14*log(x13) + p16*log(x11) + p33*log(x11
    ) - p35*log(x13) + p32*log(x22) - p34*log(x21) + p85*log(x11) - p88*log(x13) +
    p98*log(x11) - p97*log(x13) + p86*log(x30) - p87*log(x32) - (1260*x10*x12*x13*
    x24*x25*x29*x33*x34*(1/p1 + 1)*(1/p2 + 1)*(1/p3 + 1)*(1/p4 + 1)*(1/p5 + 1)*(1/p6
     + 1)*(1/p7 + 1)*(1/p8 + 1))/((x12/p3 + 1)*(x10/p7 + 1)*(x13/p8 + 1)*(x24/p1 +
    1)*(x25/p2 + 1)*(x29/p6 + 1)*(x33/p4 + 1)*(x34/p5 + 1))
```

```
d/dt(x14) = p18*log(x6) - p19*log(x8) + p17*log(x15) - p20*log(x14) + p21*log(x16)
    + p96*log(x6) - p94*log(x12) - p93*log(x14) + p95*log(x29)
d/dt(x15) = p19*log(x8) - p18*log(x6) + p23*log(x7) - p17*log(x15) + p20*log(x14) -
     p25*log(x9) - p21*log(x16) + p22*log(x16) - p24*log(x15) - p37*log(x7) + p39*
    log(x8) + p40*log(x9) - p36*log(x15) - p38*log(x21) + p42*log(x30) - p41*log(x32
    ) + p61*log(x16) - p62*log(x15) + p60*log(x22) + p59*log(x32) - p64*log(x27) -
    p63*log(x30)
d/dt(x16) = p25*log(x9) - p23*log(x7) - p22*log(x16) + p24*log(x15) - p61*log(x16)
    + p62*log(x15) - p60*log(x22) + p75*log(x8) + p76*log(x7) - p81*log(x9) - p59*
    log(x32) + p64*log(x27) + p63*log(x30) + p77*log(x19) - p80*log(x16) + p89*log(
    x8) - p82*log(x17) - p84*log(x15) - p92*log(x16) + p78*log(x31) + p79*log(x32) -
     p83*log(x30) + p90*log(x27) - p91*log(x28) - p106*log(x16) + p104*log(x27) +
    p105*log(x31) + 1
d/dt(x17) = 2*p26*log(x9) - 2*p29*log(x7) + 2*p27*log(x19) - 2*p30*log(x17) + 2*p28
    *log(x20) - 2*p31*log(x18) + 2*p75*log(x8) + 2*p76*log(x7) - 2*p72*log(x17) - 2*
    p81*log(x9) - 2*p71*log(x20) + 2*p73*log(x18) + 2*p74*log(x19) + 2*p77*log(x19)
    - 2*p80*log(x16) - 2*p82*log(x17) - 2*p84*log(x15) + 2*p78*log(x31) + 2*p79*log(
    x32) - 2*p83*log(x30) + 2
d/dt(x18) = 4*p26*log(x9) - 4*p29*log(x7) + 4*p27*log(x19) - 4*p30*log(x17) + 4*p28
    *log(x20) - 4*p31*log(x18) + 6*p72*log(x17) + 6*p71*log(x20) - 6*p73*log(x18) -
    6*p74*log(x19) - 3*p101*log(x18) + 3*p103*log(x20) - 3*p99*log(x32) - 3*p100*log
    (x31) + 3*p102*log(x30) - p115*log(x18) + p117*log(x20) + p116*log(x31) + 6
d/dt(x19) = 2*p29*log(x7) - 2*p26*log(x9) - 2*p27*log(x19) + 2*p30*log(x17) - 2*p28
    *log(x20) + 2*p31*log(x18) - 2*p75*log(x8) - 2*p76*log(x7) + 2*p72*log(x17) + 2*
    p81*log(x9) + 2*p71*log(x20) - 2*p73*log(x18) - 2*p74*log(x19) - 2*p77*log(x19)
    + 2*p80*log(x16) + 2*p82*log(x17) + 2*p84*log(x15) - 2*p78*log(x31) - 2*p79*log(
    x32) + 2*p83*log(x30) - 2
d/dt(x20) = 4*p29*log(x7) - 4*p26*log(x9) - 4*p27*log(x19) + 4*p30*log(x17) - 4*p28
    *log(x20) + 4*p31*log(x18) - 6*p72*log(x17) - 6*p71*log(x20) + 6*p73*log(x18) +
    6*p74*log(x19) + 3*p101*log(x18) - 3*p103*log(x20) + 3*p99*log(x32) + 3*p100*log
    (x31) - 3*p102*log(x30) + p115*log(x18) - p117*log(x20) - p116*log(x31) - 6
d/dt(x21) = p33*log(x11) - p37*log(x7) + p39*log(x8) - p35*log(x13) + p40*log(x9) -
     p36*log(x15) + p32*log(x22) - p34*log(x21) - p38*log(x21) + p67*log(x2) + p42*
    log(x30) - p41*log(x32) - p65*log(x21) - p66*log(x23) + p68*log(x25) - 1
d/dt(x22) = p35*log(x13) - p33*log(x11) - p32*log(x22) + p34*log(x21) - p61*log(x16
    ) + p62*log(x15) - p60*log(x22) - p59*log(x32) + p64*log(x27) - p70*log(x22) +
    p63*log(x30) + p69*log(x24) - 1
d/dt(x23) = p47*log(x11) - p48*log(x11) - p49*log(x13) + p54*log(x12) + p43*log(x25
    ) - p45*log(x23) + p58*log(x10) + p67*log(x2) + p44*log(x26) - p46*log(x24) -
    p50*log(x22) - p53*log(x23) - p51*log(x28) - p52*log(x29) + p56*log(x25) + p55*
    log(x27) - p57*log(x27) - p65*log(x21) - p66*log(x23) + p68*log(x25) + (120*x10*
    x12*x13*x24*x25*x29*x33*x34*(1/p1 + 1)*(1/p2 + 1)*(1/p3 + 1)*(1/p4 + 1)*(1/p5 +
    1)*(1/p6 + 1)*(1/p7 + 1)*(1/p8 + 1))/((x12/p3 + 1)*(x10/p7 + 1)*(x13/p8 + 1)*(
    x24/p1 + 1)*(x25/p2 + 1)*(x29/p6 + 1)*(x33/p4 + 1)*(x34/p5 + 1)) - 1
d/dt(x24) = p47*log(x11) - p48*log(x11) - p49*log(x13) + p43*log(x25) - p45*log(x23
    ) + p44*log(x26) - p46*log(x24) - p50*log(x22) + p70*log(x22) - p69*log(x24) -
    (120*x10*x12*x13*x24*x25*x29*x33*x34*(1/p1 + 1)*(1/p2 + 1)*(1/p3 + 1)*(1/p4 + 1)
    *(1/p5 + 1)*(1/p6 + 1)*(1/p7 + 1)*(1/p8 + 1))/((x12/p3 + 1)*(x10/p7 + 1)*(x13/p8
     + 1)*(x24/p1 + 1)*(x25/p2 + 1)*(x29/p6 + 1)*(x33/p4 + 1)*(x34/p5 + 1))
d/dt(x25) = p48*log(x11) - p47*log(x11) + p49*log(x13) - p54*log(x12) - p43*log(x25
    ) + p45*log(x23) - p58*log(x10) - p67*log(x2) - p44*log(x26) + p46*log(x24) +
    p50*log(x22) + p53*log(x23) + p51*log(x28) + p52*log(x29) - p56*log(x25) - p55*
    log(x27) + p57*log(x27) + p65*log(x21) + p66*log(x23) - p68*log(x25) - (120*x10*
    x12*x13*x24*x25*x29*x33*x34*(1/p1 + 1)*(1/p2 + 1)*(1/p3 + 1)*(1/p4 + 1)*(1/p5 +
    1)*(1/p6 + 1)*(1/p7 + 1)*(1/p8 + 1))/((x12/p3 + 1)*(x10/p7 + 1)*(x13/p8 + 1)*(
```

x24/p1 + 1)*(x25/p2 + 1)*(x29/p6 + 1)*(x33/p4 + 1)*(x34/p5 + 1)) + 1
d/dt(x26) = (p48*log(x11))/2 − (p47*log(x11))/2 + (p49*log(x13))/2 − (p43*log(x25))/2 + (p45*log(x23))/2 − (p44*log(x26))/2 + (p46*log(x24))/2 + (p50*log(x22))/2 + p107*log(x1) − p108*log(x26) + 1/2
d/dt(x27) = p53*log(x23) − p58*log(x10) − p54*log(x12) + p61*log(x16) − p62*log(x15) + p51*log(x28) + p52*log(x29) − p56*log(x25) − p55*log(x27) + p60*log(x22) + p57*log(x27) + p59*log(x32) − p64*log(x27) − p63*log(x30) − p89*log(x8) + p92*log(x16) − p90*log(x27) + p91*log(x28) + p106*log(x16) − p104*log(x27) − p105*log(x31)
d/dt(x28) = p54*log(x12) + p58*log(x10) − p53*log(x23) − p51*log(x28) − p52*log(x29) + p56*log(x25) + p55*log(x27) − p57*log(x27) + p89*log(x8) − p92*log(x16) + p90*log(x27) − p91*log(x28) + (120*x10*x12*x13*x24*x25*x29*x33*x34*(1/p1 + 1)*(1/p2 + 1)*(1/p3 + 1)*(1/p4 + 1)*(1/p5 + 1)*(1/p6 + 1)*(1/p7 + 1)*(1/p8 + 1))/((x12/p3 + 1)*(x10/p7 + 1)*(x13/p8 + 1)*(x24/p1 + 1)*(x25/p2 + 1)*(x29/p6 + 1)*(x33/p4 + 1)*(x34/p5 + 1))
d/dt(x29) = p54*log(x12) + p58*log(x10) − p53*log(x23) − p51*log(x28) − p52*log(x29) + p56*log(x25) + p55*log(x27) − p57*log(x27) − p96*log(x6) + p94*log(x12) + p93*log(x14) − p95*log(x29) − (120*x10*x12*x13*x24*x25*x29*x33*x34*(1/p1 + 1)*(1/p2 + 1)*(1/p3 + 1)*(1/p4 + 1)*(1/p5 + 1)*(1/p6 + 1)*(1/p7 + 1)*(1/p8 + 1))/((x12/p3 + 1)*(x10/p7 + 1)*(x13/p8 + 1)*(x24/p1 + 1)*(x25/p2 + 1)*(x29/p6 + 1)*(x33/p4 + 1)*(x34/p5 + 1))
d/dt(x30) = p61*log(x16) − p62*log(x15) + p60*log(x22) + p75*log(x8) + p76*log(x7) − p81*log(x9) + p59*log(x32) − p64*log(x27) − p63*log(x30) + p77*log(x19) − p80*log(x16) − p85*log(x11) − p82*log(x17) − p84*log(x15) + p88*log(x13) + p78*log(x31) + p79*log(x32) − p83*log(x30) − p86*log(x30) + p87*log(x32) + p101*log(x18) − p103*log(x20) + p99*log(x32) + p100*log(x31) − p102*log(x30) + 2
d/dt(x31) = p81*log(x9) − p76*log(x7) − p75*log(x8) − p77*log(x19) + p80*log(x16) + p82*log(x17) + p84*log(x15) − p78*log(x31) − p79*log(x32) + p83*log(x30) − p101*log(x18) + p106*log(x16) + p103*log(x20) − p99*log(x32) − p100*log(x31) − p104*log(x27) + p102*log(x30) + p115*log(x18) − p105*log(x31) − p117*log(x20) − p116*log(x31) − 2
d/dt(x32) = p62*log(x15) − p61*log(x16) − p60*log(x22) − p75*log(x8) − p76*log(x7) + p81*log(x9) − p59*log(x32) + p64*log(x27) + p63*log(x30) − p77*log(x19) + p80*log(x16) + p85*log(x11) + p82*log(x17) + p84*log(x15) − p88*log(x13) − p78*log(x31) − p79*log(x32) + p83*log(x30) + p86*log(x30) − p87*log(x32) − p101*log(x18) + p103*log(x20) − p99*log(x32) − p100*log(x31) + p102*log(x30) − 2
d/dt(x33) = p109*log(x3) − p110*log(x33) − (120*x10*x12*x13*x24*x25*x29*x33*x34*(1/p1 + 1)*(1/p2 + 1)*(1/p3 + 1)*(1/p4 + 1)*(1/p5 + 1)*(1/p6 + 1)*(1/p7 + 1)*(1/p8 + 1))/((x12/p3 + 1)*(x10/p7 + 1)*(x13/p8 + 1)*(x24/p1 + 1)*(x25/p2 + 1)*(x29/p6 + 1)*(x33/p4 + 1)*(x34/p5 + 1)) + 1
d/dt(x34) = p111*log(x4) − p112*log(x34) − (120*x10*x12*x13*x24*x25*x29*x33*x34*(1/p1 + 1)*(1/p2 + 1)*(1/p3 + 1)*(1/p4 + 1)*(1/p5 + 1)*(1/p6 + 1)*(1/p7 + 1)*(1/p8 + 1))/((x12/p3 + 1)*(x10/p7 + 1)*(x13/p8 + 1)*(x24/p1 + 1)*(x25/p2 + 1)*(x29/p6 + 1)*(x33/p4 + 1)*(x34/p5 + 1)) + 1

1.3.2 JAKSTAT model

```
d/dt(x1) = 2*p2*x2 + p5*x6 + p8*x7 - 2*p1*x1^2 - p4*x1*x4 - p7*x1*x5
d/dt(x2) = p3*x3 - p2*x2 + p10*x8 + p1*x1^2 - p9*x2*x4
d/dt(x3) = p31*x27 - p24*x3 - p3*x3 + p23*x14^2 - p30*x3*x16
d/dt(x4) = p5*x6 + p6*x6 + p10*x8 + p11*x8 - p4*x1*x4 - p9*x2*x4
d/dt(x5) = p6*x6 + p8*x7 - p12*x5 - p7*x1*x5
d/dt(x6) = p4*x1*x4 - p6*x6 - p5*x6
d/dt(x7) = p11*x8 - p8*x7 + p7*x1*x5
d/dt(x8) = p9*x2*x4 - p11*x8 - p10*x8
d/dt(x9) = p12*x5 + p17*x15 + p20*x23 + p22*x21 + p29*x25 + p47*x26 - p16*x9*x14 -
    p19*x9*x22 - p21*x9*x20
d/dt(x10) = (p14*x2)/(p15 + x2) - p13*x10
d/dt(x11) = p13*x10 - p50*x11
d/dt(x12) = p42*x30 - p41*x12*x29
d/dt(x13) = p26*x22 - p48*x13 + p51*x11 + p47*x26 - p25*x13*x20
d/dt(x14) = 2*p24*x3 + p17*x15 + p27*x21 + p34*x24 + p36*x25 - 2*p23*x14^2 - p16*x9
    *x14 - p35*x14*x16 - p33*x14*x20
d/dt(x15) = p28*x27 - p17*x15 + p16*x9*x14
d/dt(x16) = p29*x25 + p28*x27 + p31*x27 + p36*x25 - p30*x3*x16 - p35*x14*x16
d/dt(x17) = p39*x18*x20 - p40*x17 - p18*x17
d/dt(x18) = p18*x17 + p40*x17 + p46*x26 + p47*x26 - p39*x18*x20 - p45*x18*x23
d/dt(x19) = p18*x17 - p32*x19 - p38*x19 + p47*x26 + p37*x28^2
d/dt(x20) = p22*x21 + p26*x22 + p27*x21 + p32*x19 + p40*x17 + p34*x24 - p25*x13*x20
     - p33*x14*x20 - p39*x18*x20 - x9*x20*x21
d/dt(x21) = p21*x9*x20 - p27*x21 - p22*x21
d/dt(x22) = p20*x23 - p26*x22 - p19*x9*x22 + p25*x13*x20
d/dt(x23) = p46*x26 - p20*x23 + p19*x9*x22 - p45*x18*x23
d/dt(x24) = p33*x14*x20 - p34*x24
d/dt(x25) = p35*x14*x16 - p36*x25 - p29*x25
d/dt(x26) = p45*x18*x23 - p47*x26 - p49*x26 - p46*x26
d/dt(x27) = p30*x3*x16 - p31*x27 - p28*x27
d/dt(x28) = 2*p38*x19 - p44*x28 + p43*x30 - 2*p37*x28^2
d/dt(x29) = p42*x30 - p41*x12*x29
d/dt(x30) = p44*x28 - p42*x30 - p43*x30 + p41*x12*x29
d/dt(x31) = p49*x26
```

1.3.3 MAP Kinase model

```
d/dt(x1) = 0
d/dt(x2) = p17 + p2*x3 - p10*x2 + p11*x6 - p1*x1*x2
d/dt(x3) = 2*p3*x4 - p2*x3 - 2*p4*x3^2 + p1*x1*x2
d/dt(x4) = p6*x5 - p5*x4 - p3*x4 + p4*x3^2
d/dt(x5) = p5*x4 - p6*x5 - p12*x5 + p14*x15 - p13*x5*x14
d/dt(x6) = p10*x2 - p11*x6 + p16*x10 - p83*x6 - p15*x6*x16
d/dt(x7) = p7*x12*x23 - p9*x7 - p8*x7
d/dt(x8) = p5*x11 - p6*x8 + p12*x5 + p19*x17 - p83*x8 - p18*x8*x14
d/dt(x9) = p9*x7 - p20*x9 + p9*x88 + p9*x89 + p9*x90 + p9*x91 + p9*x92 + p9*x93 +
    p9*x94
d/dt(x10) = 2*p3*x11 - p16*x10 - 2*p4*x10^2 + p15*x6*x16
d/dt(x11) = p6*x8 - p3*x11 - p5*x11 + p4*x10^2
d/dt(x12) = p8*x7 + p20*x9 + p8*x88 + p8*x89 + p8*x90 + p8*x91 + p8*x92 + p8*x93 +
    p8*x94 - p7*x12*x23 - p7*x12*x25 - p7*x12*x27 - p7*x12*x29 - p7*x12*x34 - p7*x12
    *x35 - p7*x12*x36 - p7*x12*x37
d/dt(x13) = p84*x16
d/dt(x14) = p14*x15 + p19*x17 - p13*x5*x14 - p18*x8*x14
d/dt(x15) = p11*x17 - p10*x15 - p14*x15 + p21*x23 + p34*x32 + p49*x25 + p46*x35 +
    p55*x33 + p55*x34 + p13*x5*x14 - p22*x15*x22 - p33*x15*x31 - p50*x15*x30 - p45*
    x15*x38 - p56*x15*x39 - p56*x15*x40
d/dt(x16) = p16*x10 - p84*x16 - p15*x6*x16
d/dt(x17) = p10*x15 - p11*x17 - p19*x17 + p21*x18 + p49*x19 + p34*x63 + p46*x66 +
    p55*x64 + p55*x65 + p18*x8*x14 - p22*x17*x22 - p33*x17*x31 - p50*x17*x30 - p45*
    x17*x38 - p56*x17*x39 - p56*x17*x40
d/dt(x18) = p9*x7 + p12*x23 - p21*x18 + p23*x19 + p22*x17*x22 - p24*x18*x24
d/dt(x19) = p10*x25 - p11*x19 - p23*x19 + p25*x20 + p28*x20 + p29*x21 + p31*x21 -
    p49*x19 + p9*x88 + p24*x18*x24 - p26*x19*x26 - p32*x19*x26 + p50*x17*x30 - p27*
    x19*x69 - p30*x19*x71
d/dt(x20) = p10*x27 - p11*x20 - p25*x20 - p28*x20 + p9*x89 + p26*x19*x26 + p27*x19*
    x69
d/dt(x21) = p10*x29 - p11*x21 - p29*x21 - p31*x21 + p9*x90 + p32*x19*x26 + p30*x19*
    x71
d/dt(x22) = p21*x18 + p21*x23 + p38*x34 + p38*x39 + p51*x30 + p38*x65 - p22*x15*x22
     - p22*x17*x22 - p37*x22*x33 - p52*x22*x24 - p37*x22*x40 - p37*x22*x64
d/dt(x23) = p8*x7 - p12*x23 - p21*x23 + p23*x25 - p7*x12*x23 + p22*x15*x22 - p24*
    x23*x24
d/dt(x24) = p23*x19 + p23*x25 + p39*x35 + p51*x30 + p58*x38 + p39*x66 - p24*x18*x24
     - p24*x23*x24 - p40*x24*x34 - p52*x22*x24 - p57*x24*x39 - p40*x24*x65
d/dt(x25) = p11*x19 - p10*x25 - p23*x25 + p25*x27 + p28*x27 + p29*x29 + p31*x29 -
    p49*x25 + p8*x88 - p7*x12*x25 + p24*x23*x24 - p26*x25*x26 - p27*x25*x28 - p32*
    x25*x26 + p50*x15*x30 - p30*x25*x43
d/dt(x26) = p25*x20 + p25*x27 + p31*x21 + p31*x29 + p25*x36 + p31*x37 + p25*x67 +
    p31*x68 - p26*x19*x26 - p26*x25*x26 - p32*x19*x26 - p32*x25*x26 - p26*x26*x35 -
    p32*x26*x35 - p26*x26*x66 - p32*x26*x66
d/dt(x27) = p11*x20 - p10*x27 - p25*x27 - p28*x27 + p8*x89 - p7*x12*x27 + p26*x25*
    x26 + p27*x25*x28
d/dt(x28) = p28*x27 + p28*x36 + p42*x42 - p27*x25*x28 - p27*x28*x35 - p41*x28*x41
d/dt(x29) = p11*x21 - p10*x29 - p29*x29 - p31*x29 + p8*x90 - p7*x12*x29 + p32*x25*
    x26 + p30*x25*x43
d/dt(x30) = p49*x19 + p49*x25 - p51*x30 + p47*x38 + p59*x35 + p59*x66 - p50*x15*x30
     - p50*x17*x30 + p52*x22*x24 - p48*x30*x40 - p60*x30*x33 - p60*x30*x64
d/dt(x31) = p34*x32 + p34*x63 - p33*x15*x31 - p33*x17*x31 + (p53*x40)/(p54 + x40)
d/dt(x32) = p36*x33 - p34*x32 - p35*x32 - p10*x32 + p11*x63 + p33*x15*x31
```

```
d/dt(x33) = p35*x32 - p10*x33 - p36*x33 + p38*x34 + p11*x64 - p55*x33 + p59*x35 -
    p37*x22*x33 + p56*x15*x40 - p60*x30*x33
d/dt(x34) = p39*x35 - p38*x34 - p10*x34 + p11*x65 - p55*x34 + p8*x91 - p7*x12*x34 +
     p37*x22*x33 - p40*x24*x34 + p56*x15*x39
d/dt(x35) = p25*x36 - p10*x35 + p28*x36 + p29*x37 + p31*x37 - p39*x35 + p11*x66 -
    p46*x35 - p59*x35 + p8*x92 - p7*x12*x35 - p26*x26*x35 - p27*x28*x35 - p32*x26*
    x35 + p40*x24*x34 + p45*x15*x38 - p30*x35*x43 + p60*x30*x33
d/dt(x36) = p11*x67 - p25*x36 - p28*x36 - p10*x36 + p8*x93 - p7*x12*x36 + p26*x26*
    x35 + p27*x28*x35
d/dt(x37) = p11*x68 - p29*x37 - p31*x37 - p10*x37 + p8*x94 - p7*x12*x37 + p32*x26*
    x35 + p30*x35*x43
d/dt(x38) = p46*x35 - p47*x38 - p58*x38 + p46*x66 - p45*x15*x38 - p45*x17*x38 + p48
    *x30*x40 + p57*x24*x39
d/dt(x39) = p55*x34 - p38*x39 + p58*x38 + p55*x65 + p37*x22*x40 - p56*x15*x39 - p56
    *x17*x39 - p57*x24*x39
d/dt(x40) = p38*x39 + p47*x38 + p55*x33 + p55*x64 - p37*x22*x40 - p56*x15*x40 - p56
    *x17*x40 - p48*x30*x40 - (p53*x40)/(p54 + x40)
d/dt(x41) = p42*x42 + p63*x46 + p42*x70 + p63*x73 - p41*x28*x41 - p41*x41*x69
d/dt(x42) = p41*x28*x41 - p43*x42 - p42*x42 + p44*x43*x45
d/dt(x43) = p29*x29 + p29*x37 + p43*x42 - p30*x25*x43 - p30*x35*x43 - p44*x43*x45
d/dt(x44) = p62*x46 + p63*x46 + p62*x73 + p63*x73 - p61*x44*x45 - p61*x44*x72
d/dt(x45) = p43*x42 + p62*x46 + p64*x48 + p64*x50 + p66*x48 + p67*x50 - p44*x43*x45
     - p61*x44*x45 - p65*x45*x47 - p65*x45*x49
d/dt(x46) = p61*x44*x45 - p63*x46 - p62*x46
d/dt(x47) = p64*x48 + p70*x54 + p64*x74 + p70*x79 - p65*x45*x47 - p65*x47*x72
d/dt(x48) = p65*x45*x47 - p66*x48 - p64*x48
d/dt(x49) = p64*x50 + p66*x48 + p70*x52 + p71*x54 - p65*x45*x49 - p72*x49*x53
d/dt(x50) = p65*x45*x49 - p67*x50 - p64*x50
d/dt(x51) = p67*x50 + p68*x52 + p74*x56 + p75*x56 + p74*x58 + p76*x58 - p69*x51*x53
     - p73*x51*x55 - p73*x51*x57
d/dt(x52) = p69*x51*x53 - p70*x52 - p68*x52
d/dt(x53) = p68*x52 + p70*x52 + p70*x54 + p71*x54 + p68*x78 + p70*x78 + p70*x79 +
    p71*x79 - p69*x51*x53 - p72*x49*x53 - p69*x53*x77 - p72*x53*x75
d/dt(x54) = p72*x49*x53 - p71*x54 - p70*x54
d/dt(x55) = p74*x56 + p82*x62 + p74*x80 + p82*x85 - p73*x51*x55 - p73*x55*x77
d/dt(x56) = p73*x51*x55 - p75*x56 - p74*x56
d/dt(x57) = p75*x56 + p74*x58 + p79*x61 + p80*x62 - p73*x51*x57 - p81*x57*x60
d/dt(x58) = p73*x51*x57 - p76*x58 - p74*x58
d/dt(x59) = p76*x58 + p77*x61 - p78*x59*x60
d/dt(x60) = p77*x61 + p79*x61 + p80*x62 + p82*x62 + p77*x84 + p79*x84 + p80*x85 +
    p82*x85 - p78*x59*x60 - p81*x57*x60 - p78*x60*x83 - p81*x60*x81
d/dt(x61) = p78*x59*x60 - p79*x61 - p77*x61
d/dt(x62) = p81*x57*x60 - p82*x62 - p80*x62
d/dt(x63) = p10*x32 - p11*x63 - p34*x63 - p35*x63 + p36*x64 + p33*x17*x31
d/dt(x64) = p10*x33 - p11*x64 + p35*x63 - p36*x64 + p38*x65 - p55*x64 + p59*x66 +
    p56*x17*x40 - p37*x22*x64 - p60*x30*x64
d/dt(x65) = p10*x34 - p11*x65 + p9*x91 - p38*x65 + p39*x66 - p55*x65 + p56*x17*x39
    + p37*x22*x64 - p40*x24*x65
d/dt(x66) = p10*x35 - p11*x66 + p25*x67 + p28*x67 + p29*x68 + p31*x68 + p9*x92 -
    p39*x66 - p46*x66 - p59*x66 + p45*x17*x38 - p26*x26*x66 - p32*x26*x66 + p40*x24*
    x65 + p60*x30*x64 - p27*x66*x69 - p30*x66*x71
d/dt(x67) = p10*x36 - p11*x67 - p25*x67 - p28*x67 + p9*x93 + p26*x26*x66 + p27*x66*
    x69
d/dt(x68) = p10*x37 - p11*x68 - p29*x68 - p31*x68 + p9*x94 + p32*x26*x66 + p30*x66*
    x71
```

```
d/dt(x69) = p28*x20 + p28*x67 + p42*x70 - p27*x19*x69 - p41*x41*x69 - p27*x66*x69
d/dt(x70) = p41*x41*x69 - p43*x70 - p42*x70 + p44*x71*x72
d/dt(x71) = p29*x21 + p29*x68 + p43*x70 - p30*x19*x71 - p30*x66*x71 - p44*x71*x72
d/dt(x72) = p43*x70 + p62*x73 + p64*x74 + p64*x76 + p66*x74 + p67*x76 - p61*x44*x72
    - p65*x47*x72 - p44*x71*x72 - p65*x72*x75
d/dt(x73) = p61*x44*x72 - p63*x73 - p62*x73
d/dt(x74) = p65*x47*x72 - p66*x74 - p64*x74
d/dt(x75) = p64*x76 + p66*x74 + p70*x78 + p71*x79 - p72*x53*x75 - p65*x72*x75
d/dt(x76) = p65*x72*x75 - p67*x76 - p64*x76
d/dt(x77) = p67*x76 + p68*x78 + p74*x80 + p75*x80 + p74*x82 + p76*x82 - p69*x53*x77
    - p73*x55*x77 - p73*x77*x81
d/dt(x78) = p69*x53*x77 - p70*x78 - p68*x78
d/dt(x79) = p72*x53*x75 - p71*x79 - p70*x79
d/dt(x80) = p73*x55*x77 - p75*x80 - p74*x80
d/dt(x81) = p75*x80 + p74*x82 + p79*x84 + p80*x85 - p81*x60*x81 - p73*x77*x81
d/dt(x82) = p73*x77*x81 - p76*x82 - p74*x82
d/dt(x83) = p76*x82 + p77*x84 - p78*x60*x83
d/dt(x84) = p78*x60*x83 - p79*x84 - p77*x84
d/dt(x85) = p81*x60*x81 - p82*x85 - p80*x85
d/dt(x86) = p83*x6
d/dt(x87) = p83*x8
d/dt(x88) = p7*x12*x25 - p9*x88 - p8*x88
d/dt(x89) = p7*x12*x27 - p9*x89 - p8*x89
d/dt(x90) = p7*x12*x29 - p9*x90 - p8*x90
d/dt(x91) = p7*x12*x34 - p9*x91 - p8*x91
d/dt(x92) = p7*x12*x35 - p9*x92 - p8*x92
d/dt(x93) = p7*x12*x36 - p9*x93 - p8*x93
d/dt(x94) = p7*x12*x37 - p9*x94 - p8*x94
d/dt(x95) = x45 + x46 + x48 + x50 + x72 + x73 + x74 + x76
d/dt(x96) = x28 + x42 + x69 + x70
d/dt(x97) = x51 + x77
d/dt(x98) = x59 + x83
d/dt(x99) = x33 + x34 + x35 + x36 + x37 + x38 + x39 + x40 + x64 + x65 + x66 + x67 +
    x68 + x91 + x92 + x93 + x94
d/dt(x100) = x5 + x7 + x8 + x11 + x15 + x17 + x18 + x19 + x20 + x21 + x23 + x25 +
   x27 + x29 + x32 + x33 + x34 + x35 + x36 + x37 + x63 + x64 + x65 + x66 + x67 +
   x68 + x88 + x89 + x90 + x91 + x92 + x93 + x94
```

References

1. Chappell, M. & Gunn, R. A procedure for generating locally identifiable reparameterisation of unidentfiable non-linear systems by the similarity transformation approach. *Math. Biosci.* **148**, 21 (1998).

2. Stigter, J. & Molenaar, J. A fast algorithm to assess local structural identifiability. *Autom.* **58**, 118–124 (2015).

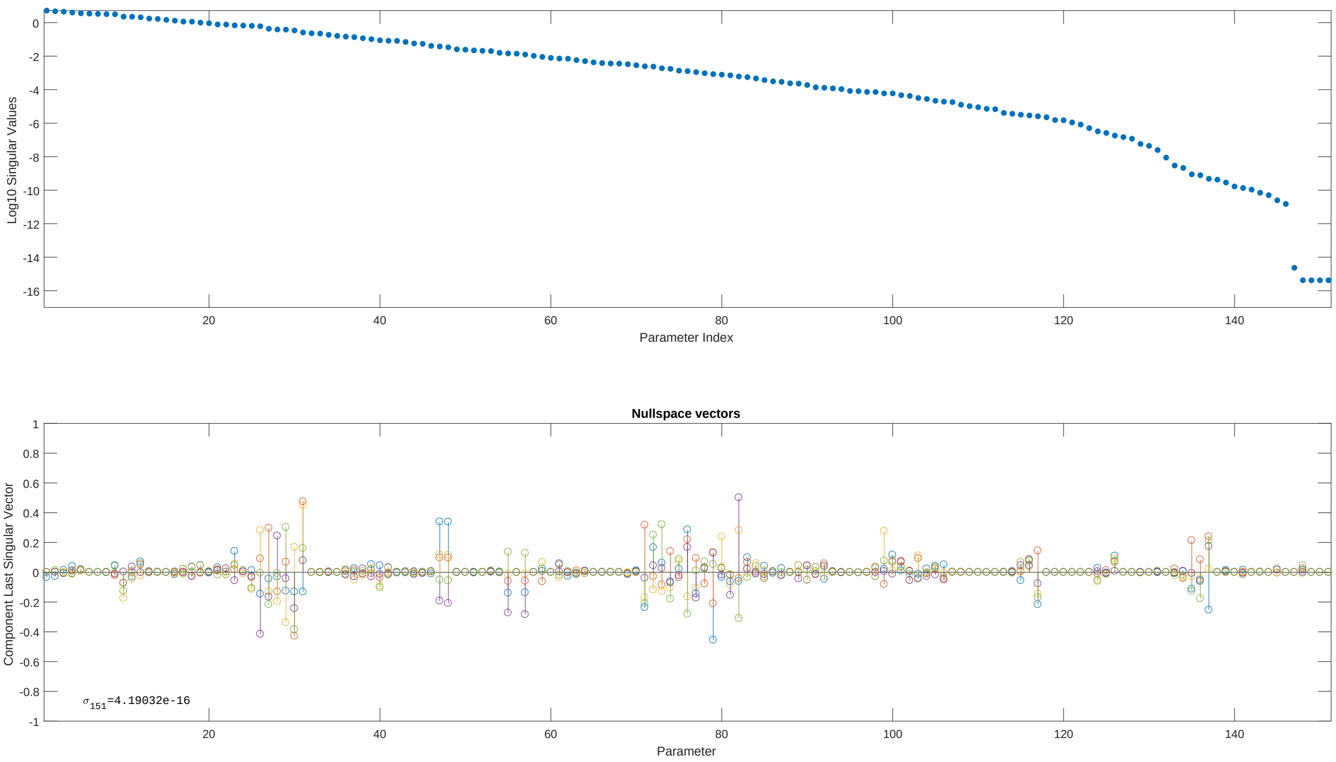

Supplementary Figure 1. Observability signature Chinese Hamster model based on one trial $S(t_0,\ldots,t_N,\theta^1)$. The top graph shows all singular values for the 151 parameters in the model (including the parametrized initial conditions). The bottom graph shows all nullspace vectors $v_{147}-v_{151}$ in V corresponding to the 5 smallest singular values (order 10^{-16}) that appear after the gap. Apparently, using only one trial θ^1 overestimates the number of zero-singular-values due to inaccuracies in the computation and/or a lack of excitation of the parametric output sensitivities. Although a gap is clearly visible, the nullspace graph shows that the true dependencies between the four parameters as in figure **??** are hidden because of inaccuracies.
